# Acute Effects of Air Pollution on Ischemic Heart Disease Hospitalizations: A Population-Based Time-Series Study in Wuhan, China, 2017–2018

**DOI:** 10.3390/ijerph182312527

**Published:** 2021-11-28

**Authors:** Wanglin Xu, Xingyuan Liu, Zenghui Huang, Yating Du, Biao Zhang, Qiaomai Wang, Jing Xiang, Yuliang Zou, Lu Ma

**Affiliations:** 1 School of Public Health, Wuhan University, Wuhan 430071, China; wanglin.xu@outlook.com (W.X.); huangzenghui@whu.edu.cn (Z.H.); 2015302170044@whu.edu.cn (Y.D.); zbiao108@163.com (B.Z.); 2020283050043@whu.edu.cn (Q.W.); 2020303051070@whu.edu.cn (J.X.); 2 Information Center of Health and Family Planning, Wuhan 430021, China; lxyxxzxwhs@163.com

**Keywords:** air pollution, ischemic heart disease, hospitalization, time-series study, Wuhan

## Abstract

Evidence of the acute effects of air pollutants on ischemic heart disease (IHD) hospitalizations based on the entire population of a megacity in central China is lacking. All IHD hospitalization records from 2017 to 2018 were obtained from the Wuhan Information Center of Health and Family Planning. Daily air pollutant concentrations and meteorological data were synchronously collected from the Wuhan Environmental Protection Bureau. A time-series study using generalized additive models was conducted to systematically examine the associations between air pollutants and IHD hospitalizations. Stratified analyses by gender, age, season, hypertension, diabetes, and hyperlipidemia were performed. In total, 139,616 IHD hospitalizations were included. Short-term exposure to air pollutants was positively associated with IHD hospitalizations. The age group ≥76 was at higher exposure risk, and the associations appeared to be more evident in cold seasons. PM_2.5_ and PM_10_ appeared to have greater effects on males and those without hypertension or diabetes, whereas NO_2_ and SO_2_ had greater effects on females and those with hypertension or diabetes. The risk of IHD hospitalization due to air pollutants was greater in people without hyperlipidemia. Our study provides new evidence of the effects of air pollution on the increased incidence of IHD in central China.

## 1. Introduction

Ischemic heart disease (IHD) is characterized by the narrowing of coronary vessels, which supply blood to the heart muscle; it results in myocardial ischemia, and is one of the primary causes of heart failure among various non-communicable diseases [1]. IHD is the leading cause of deaths worldwide; it was responsible for 8.9 million deaths in 2019, which accounts for 16% of total deaths in the world, and the number of deaths from IHD is increasing rapidly (by more than 2 million from 2000 to 2019, according to WHO) [2]. China is facing a great challenge in IHD, in which IHD is the leading cause of disease burden, with a disability-adjusted of life years (DALY) rate of 2131/100,000 in 2017 [3]. The total cost of IHD hospitalization was 111,982 million yuan in 2018, which was the highest among all cardiovascular diseases [4].

Previous studies have provided strong epidemiologic evidence of the deleterious effects of ambient air pollutants, including PM_2.5_ [1,5,6], PM_10_ [7,8], NO_2_ [9], and SO_2_ [10] on increased cardiovascular morbidity and mortality over the last few decades. As a major public health concern for cardiovascular endpoints, IHD has received increasing attention, which has led to investigations on the relationship between air pollution and IHD [11,12]. Compelling evidence from developed countries shows increased IHD events following acute exposure to air pollutants. For example, a study conducted in the United States on fine particulate air pollution and hospital admission for cardiovascular diseases observed a 0.44% (95% confidence interval (CI) 0.02%–0.86%) increase in risk of IHD hospital admissions per 10 μg/m^3^ increase in same-day PM_2.5_ [13]. Another study in the United States reported that exposure to higher NO_2_ levels contributes to an additional 7000 non-White deaths from IHD per year compared with White people [14]. However, the results of different studies are inconsistent. For example, previous research in Hong Kong did not observe associations between PM_10_ and IHD hospitalizations or mortality [15]. Therefore, the relationship between different air pollutants and the risk of IHD hospitalization remains unclear.

In China, studies on the effects of air pollution on cardiovascular disease were more often conducted in highly industrialized northern cities with high air pollution levels, such as Beijing [16] and Shenyang [17], and only a few studies have focused on IHD. An analysis of particulate air pollution and risk of IHD hospitalization in the eastern coastal city of Shanghai, China found that each 10 μg/m^3^ increment in PM_2.5_ and PM_10_ was associated with an increase in IHD hospitalizations by 0.25% (95% CI 0.10%–0.39%) and 0.57% (95% CI 0.46%–0.68%), respectively [7]. However, the results may not be applicable to central China because of local climate conditions, chemical components, and population susceptibility. For example, the unique sea-land breeze circulation in coastal cities may result in different air pollution exposure patterns than that in inland areas [18].

Wuhan is the largest city in central China, and there have been several studies focusing on the relationship between air pollutants and cardiovascular disease there [5,19]. However, some of these studies were limited to a particular district or a hospital [19] in Wuhan, and the populations of these studies may not represent the entire population. In addition, recent large-scale studies that focused solely on the health hazards of particulate matter (PM_2.5_ and PM_10_) [5], and the relationship of ambient NO_2_ and SO_2_ with the risk of IHD hospitalization have not been adequately investigated in China [20]. Therefore, a study on the association between air pollutants (both particulate matter and gaseous pollutants) and IHD hospitalization based on the entire population of Wuhan is needed to better understand the actual impact of ambient air pollution problems in central China. The objective of this study was to explore the associations between ambient air pollutants (PM_2.5_, PM_10_, NO_2_, and SO_2_) and IHD (measured by counts of hospitalizations), based on a dataset of all hospitals in Wuhan. Further, in order to investigate whether some chronic diseases (hypertension, diabetes, and hyperlipidemia), which are increasing year by year in China and also present as important risk factors for IHD [21], change the pattern of air pollution effects, we also performed a stratified analysis of these comorbidities.

## 2. Materials and Methods

### 2.1. Study Area

Wuhan (29.58° N to 31.22° N, 113.41° E to 115.05° E), located in the eastern part of Hubei Province, inland China, covers an area of 8594 square kilometers, and has a permanent population of 12 million at the end of 2020. The terrain of Wuhan is high in the east and low in the west, and high in the south and low in the north. Due to its subtropical monsoon climate, Wuhan has typical weather characteristics with distinct seasons and abundant rainfall. As an industrial city, Wuhan is the most important industrial center in central China, which results in relatively severe air pollution.

### 2.2. Hospitalization Data

Daily hospitalization records were obtained from the Wuhan Information Center of Health and Family Planning (http://wjw.wuhan.gov.cn/ accessed on 10 September 2021) between 1 January 2017 and 31 December 2018, where all hospitals in Wuhan report information on their health care services and management. Fifty-three grade 2 or 3 hospitals with the capability to diagnose and treat patients with IHD were included in this study. The dataset consists of the primary diagnosis and other diagnoses made by specialist physicians according to the International Classification of Diseases (10th Revision), as well as basic information, including gender and age. Patients with IHD were identified by the primary diagnosis for ischemic heart disease (I20–I25). We obtained comorbidities from other diagnoses with codes I10–I15 for hypertension, E10–E14 for diabetes, and E78.0–E78.5 for hyperlipidemia to examine whether the three chronic diseases exacerbated relative risk. Daily counts of IHD hospitalizations were stratified by gender (male, female), age groups (≤65, 66–75, and ≥76 years), hypertension (with hypertension, without hypertension), diabetes (with diabetes, without diabetes), and hyperlipidemia (with hyperlipidemia, without hyperlipidemia). This study was considered exempt from institutional review board approval because the data used were collected for administrative purposes, and did not contain any personally identifiable information.

### 2.3. Air Pollution and Meteorological Data

**Figure 1:**
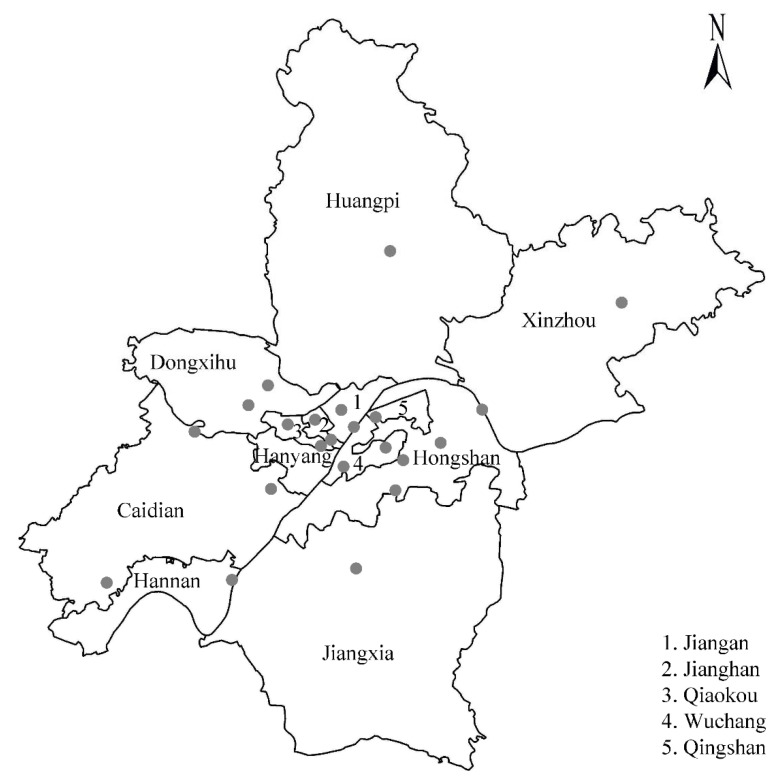
Location of the 22 air quality monitoring stations in Wuhan.

Air pollution data were collected from Wuhan Environmental Protection Bureau (http://hbj.wuhan.gov.cn/ accessed on 10 September 2021) during the study period. The database included 24-h average measurements of PM_2.5_, PM_10_, NO_2_, and SO_2_ concentrations based on the assessment of 22 air quality monitoring stations in all 13 districts of Wuhan City (Figure 1). According to construction norms for air quality monitoring stations, these sites are mandated to be located away from major roads, industrial sources, and other major emissions. Our results therefore reflect the background levels of urban air pollution in Wuhan. In addition, daily meteorological data, including daily mean temperature and relative humidity, during the study period were collected from the China National Meteorological Science Data Center (http://data.cma.cn/ accessed on 10 September 2021).

### 2.4. Statistical Analysis

Frequency and percentage were utilized to describe the study population characteristics. Air pollution, meteorological data, and daily case were described as mean ± standard deviation (SD) for continuous variables. Given that the associations between explanatory variables and hospitalizations are non-linear, generalized additive models (GAMs) with quasi-Poisson regression were applied to explore the association between air pollutants and daily IHD hospitalizations. The core model of the present study was adjusted for several covariates, and constructed as follows:

(1) $$\mathrm{Log}\left[E\left(y_{t}\right)\right]=\alpha +\beta_{1}x_{i}+ns\left(\mathrm{Temperature},\;df\right)+ns\left(\mathrm{Humidity},\;df\right)+\;ns\left(\mathrm{Time},df\right)+\beta_{2}\mathrm{factor}\left(\mathrm{DOW}\right)+\beta_{3}\mathrm{factor}\left(\mathrm{Holiday}\right),$$

where *E*[(*y_t_*)] represents the expected count of IHD hospitalizations at day *t*; *α* is the intercept value; *β*_1_, *β*_2_, and *β*_3_ are regression correlations; *x_i_* is the daily air pollutant concentration; *df* is the degrees of freedom; Temperature is daily average temperature (*df* = 3); Humidity is the daily relative humidity (*df* = 3); DOW is a dummy variable for the day of the week; Holiday indicates a public holiday in China; and ns represents a smoothed function of viable time (*df* = 4) [22,23].

We used different lag periods to estimate the lag effect associated with air pollutants, including single-day (lag0 to lag3), and moving average exposure of multiple days (lag01 to lag03). For example, lag1 represents the previous day’s concentration, and lag01 represents the average of the current and previous day’s concentrations. In addition, we conducted subgroup analyses by gender (male, female), age (≤65, 65–75, and ≥76 years), season (warm: May to October; cold: November to April), hypertension status (with hypertension, without hypertension), diabetes status (with diabetes, without diabetes), and hyperlipidemia status (with hyperlipidemia, without hyperlipidemia) to explore the potential effect of modifiers on the associations of PM_2.5_, PM_10_, NO_2_, and SO_2_ levels with cause-specific IHD hospitalizations. The exposure–response relationship between air pollutants and IHD hospitalizations was further examined by smoothing the air pollutant terms using the natural cubic spline function (*df* = 3) [24].

The results of model estimates were reported as excess risks (ERs) and 95% confidence intervals (CIs), which indicate a percentage change in daily IHD hospitalizations per 10 μg/m^3^ increase in PM_2.5_, PM_10_, NO_2_, and SO_2_ concentrations (ER = [RR − 1] × 100%). All of the above analyses were carried out by R software (version 4.1.1) (R Core Team, Vienna, Austria). *p* < 0.05 was considered statistically significant.

## 3. Results

### 3.1. Descriptive Analysis

The characteristics of the study population are summarized in Table 1. A total of 139,616 IHD hospitalizations from 1 January 2017 to 31 December 2018 in Wuhan with an average age of 68.56 years were included in the analysis. Among which, 55.7% were male, and 51.4% were admitted to hospitals during cold seasons. The mean number of daily IHD hospitalizations was 192.60 with a median of 189.00 and a range of 47.00–456.00 (Table 2). Furthermore, we found that 63.3%, 24.8%, and 17.8% of the patients with IHD had hypertension, diabetes, and hyperlipidemia, respectively.

The distributions of daily air pollutant concentrations and weather variables in Wuhan during the study period are summarized in Table 2. The daily mean concentrations of air pollutants were 48.43 μg/m^3^ for PM_2.5_, 82.07 μg/m^3^ for PM_10_, 42.22 μg/m^3^ for NO_2_, and 9.78 μg/m^3^ for SO_2_. PM_2.5_ and PM_10_ concentrations of most days were within the China ambient air quality standards (AQS) Grade II criteria, but exceeded the China AQS Grade I criteria. NO_2_ and SO_2_ concentrations were within these two criteria on almost all days (Table A1). The daily average temperature and humidity were 17.52 °C and 79.39%, respectively, which reflect the subtropical climate in Wuhan.

### 3.2. Statistical Model Analysis

Table 3 presents the ERs and 95% CIs for IHD hospitalization of air pollutant concentrations (per 10 μg/m^3^ increase) in single-lag models (lag0 to lag3) and cumulative exposure models (lag01 to lag03). IHD hospitalization had positive associations with PM_2.5_, PM_10_, NO_2_, and SO_2_ in both model types. In single-lag models, the strongest association for IHD hospitalization due to PM_2.5_, PM_10_, NO_2_, and SO_2_ occurred at the current day (lag0), with ERs of 0.50 (95% CI 0.27–0.73), 0.50 (95% CI 0.36–0.64), 2.86 (95% CI 2.45–3.26), and 3.82 (95% CI 2.17–5.49), respectively. In cumulative exposure models, the largest effects were observed at lag01 for PM_2.5_, PM_10_, and NO_2_ and lag02 for SO_2_, with ERs of 0.31 (95% CI 0.18–0.44), 0.55 (95% CI 0.41–0.70), 2.76 (95% CI 2.31–3.20), and 2.20 (95% CI 0.41–4.02), respectively.

### 3.3. Stratified Analysis

**Figure 2:**
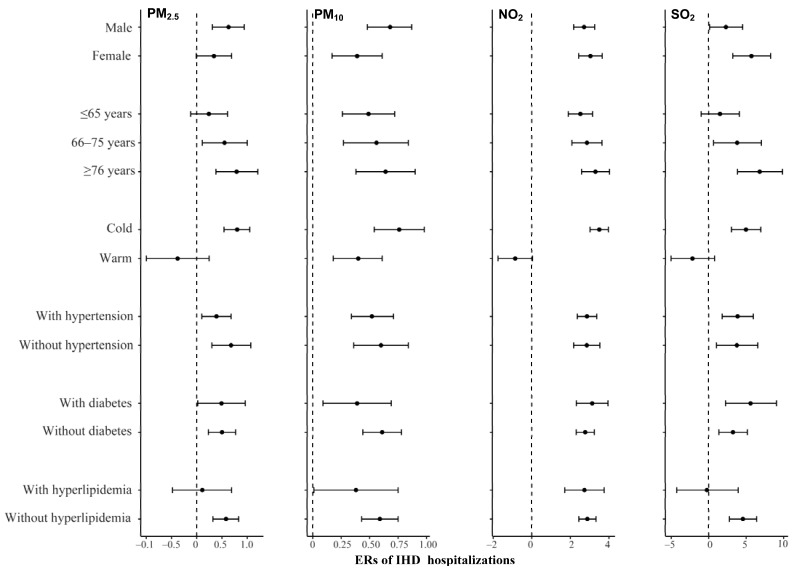
Excess risks (%) and 95% CIs of IHD hospitalization associated with per 10 μg/m^3^ increase in PM_2.5_, PM_10_, NO_2_, and SO_2_ concentrations at lag0, lag01, lag0, and lag0 day, respectively, stratified by gender, age, season, hypertension, diabetes, and hyperlipidemia.

We examined the associations between air pollution and IHD hospitalizations at lag0 for PM_2.5_, NO_2_, and SO_2_ and lag01 for PM_10_, which were classified by gender, age, season, hypertension, diabetes, and hyperlipidemia for subgroup analyses. In gender-specific analyses, IHD hospitalizations in male and female groups had positive associations with PM_10_ and NO_2_. The risk of IHD hospitalization for PM_10_ among males was higher than that in females, but the opposite was true for NO_2_. In age-specific analyses, the strongest effects of PM_2.5_, PM_10_, NO_2_, and SO_2_ on IHD hospitalization were observed in the elderly (≥76 years). In season-specific analyses, we found that PM_2.5_, PM_10_, NO_2_, and SO_2_ had greater effects on IHD hospitalizations during cold seasons than during warm seasons. In addition, the risk of hospitalization of IHD with hypertension or diabetes due to SO_2_ and NO_2_ was greater than that of IHD without hypertension or diabetes. Interestingly, the risk of hospitalization for IHD with hypertension or diabetes as a comorbidity due to PM_2.5_ and PM_10_ exposure was lower than that for IHD without hypertension or diabetes. Positive associations were found in group of IHD hospitalizations without hyperlipidemia for PM_2.5_, PM_10_, and SO_2_, and the risk of hospitalization for IHD with hyperlipidemia due to NO_2_ was lower than that without hyperlipidemia (Figure 2).

### 3.4. Exposure-Response Analysis

**Figure 3:**
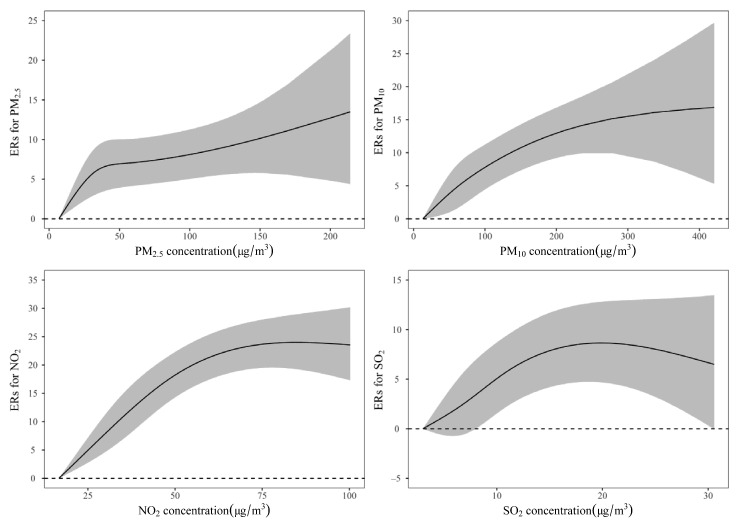
Concentration-response (E-R) curves for PM_2.5_, PM_10_, NO_2_, and SO_2_ concentrations with excess risks (%) of IHD hospitalizations at lag0, lag01, lag0, and lag0 day, respectively (*df* = 3).

We performed a dose–effect analysis at the lag days where PM_2.5_, PM_10_, NO_2_, and SO_2_ had the strongest association with IHD hospitalization, that is, at lag0, lag01, lag0, and lag0, respectively. As shown in Figure 3, a clear dose–effect relationship exists between all the four different air pollutants and IHD hospitalization. PM_2.5_ represented a steep response at lower concentrations, which tended to change linearly as the concentration increased. The result of PM_10_ exhibited a relatively large fluctuation at lower concentrations, and reached a plateau at high concentrations. The performance of NO_2_ and SO_2_ at low concentrations was similar to that of PM_10_, but a decreasing trend was observed at high concentrations.

## 4. Discussion

In this large population-based study, we found that the increase in IHD hospitalizations strongly coincided with the increase in PM_2.5_, PM_10_, NO_2_, and SO_2_ concentrations in Wuhan in 2017–2018. The results of subgroup analyses suggest that gender, age, season, and comorbidities may alter the effect. People in different gender groups were found to have different sensitivities to air pollutants; risk estimates were higher in older individuals, and during cold seasons. The relationship estimates for subgroups with and without comorbidities (including hypertensive, diabetes, and hyperlipidemia) varied from each other. We believe that this study is the most detailed study to date that has evaluated the acute impact of air pollution on IHD hospitalization based on the entire population of a major city in central China.

**Figure A1:**
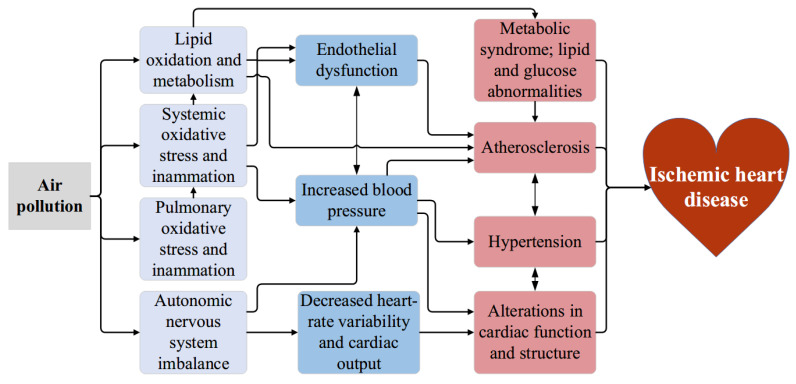
Description of the proposed physio-pathological processes between air pollution and IHD. Figure adapted from Cosselman et al. [62].

Our study found that short-term exposure to PM_2.5_, PM_10_, NO_2_, and SO_2_ was positively correlated with IHD hospitalization. This result is consistent with previous studies on air pollution and cardiovascular diseases [23,25]. The estimates for all air pollutants peaked at the current day (lag0) in single-lag models, which is in line with previous studies [26,27], and suggests that the risk of IHD hospitalization from exposure to air pollutants may occur within hours. A study in Japan found that exposure to suspended particulate matter within 6 h before the case events is associated with the risk of cardiovascular disease onset, with an odds ratio of 1.04 (95% CI 1.01–1.06) after an interquartile range increase in pollutant concentration. Another study in Germany reported that an interquartile range increase in ultrafine particles was associated with a 3.27% (95% CI 0.27–6.37) increase in myocardial infarction 6 h later. Multiple biological and physiological mechanisms could help explain the link between air pollution and IHD risk. Air pollution exposure may bring about a range of acute complications that increase cardiovascular burden and IHD risk: it may accelerate the progression of atherosclerosis; reduce plaque stability; promote acute ischemic events; decrease oxygen saturation; and cause hypoxemia [24,26,27,28,29]. In more detail, inhaled pollutants can cause oxidative stress and inflammation, leading to various tissue and organ responses, such as endothelial dysfunction, and further leading to subclinical effects, such as atherosclerosis and, ultimately, to IHD (Figure A1). A study conducted in Beijing reported a significant increase in the circulating biomarkers of plaque vulnerability from 8.6% (95% CI 0.1–17.8) to 141.4% (95% CI 111.8–171.0), which was associated with an interquartile range increase in the moving averages of PM_2.5_ during the last 1–7 days before each participant’s clinic visit [30]. Together, these findings demonstrated the immediate effect of air pollution on disease onset, although time scales may vary depending on the type of disease, the composition of air pollution, and the study design.

Our results indicated that the ERs for gaseous pollutants (NO_2_: 2.86 (95% CI 2.45–3.26) and SO_2_: 3.82 (95% CI 2.17–5.49)) were higher than those for particulate matter (PM_2.5_: 0.50 (95% CI 0.27–0.73) and PM_10_: 0.55 (95% CI 0.41–0.70)). Similar results were found in a study on the relationship between ambient air pollution and hospital admission, which found that gaseous pollutants (SO_2_ and NO_2_) had a stronger effect on cardiovascular disease hospitalization than PM_10_ [31]. A study conducted in south China reported that the burden of ischemic heart disease (years of life lost) from gaseous pollutants (NO_2_ and SO_2_) was higher than that of PM_2.5_ [32]. Although the mechanisms for the relatively higher risk due to gaseous pollutants are still undergoing continual refinement, differences in the effects of these two types of pollutants on endothelial function, which was identified by recent studies, may partially explain this phenomenon [9]. Another possible explanation is that gaseous pollutants, including NO_2_ and SO_2_, have oxidative properties, and their ability to induce oxidative stress is stronger than PM [33,34]. Emerging evidence on the associations of NO_2_ and SO_2_ with cardiovascular disease in China suggests that the health impacts of air pollution are not limited to particulate matter. Therefore, the role of exposure to ambient gaseous pollutants should be studied further.

This study explored the demographic-specific associations between air pollution and IHD hospitalization. Similar to other research [35,36], our results show that the effects of air pollution from each of the pollutants are more detrimental for older individuals. A recent cohort study in the United States suggested that the effects of exposures to air pollutants on coronary arteries might be greater in the elderly (≥65) [36]. Such an association is widely accepted because of the frailer immune systems and pre-existing chronic medical problems of the elderly [35]. The higher risk in older people may also be attributed to the cumulative toxic effects of long-term exposure to ambient pollution [37]. Therefore, older adults should be informed of the negative consequences of air pollution, and avoid exposure to contaminated air.

The assessment of gender differences in the epidemiology of air pollution has been of wide interest. In the current study, statistically significant gender differences were observed in air pollution-related IHD hospitalization hazards. Higher risk estimates were found in the male group for PM_2.5_ and PM_10_ exposure. Similar results were found in previous studies [7,38]. A population-based study conducted in Canada on the relationship between air pollution exposure and the incidence of acute myocardial infarction suggested that males might be more susceptible to PM_2.5_-related IHD hospitalizations [39]. A possible explanation for this finding might be the fact that males are more likely to be exposed to PM_2.5_ and PM_10_ because of their higher rate of outside employment compared with females [40]. On the contrary, females were more sensitive to NO_2_ and SO_2_ in our study, which is in line with several previous studies [14,30,41,42]. A study conducted in southern China showed that the risk of IHD death from short-term exposure to SO_2_ was greater in women than in men [20]. A recent study in Canada reported that females were at higher risks of IHD hospitalization from NO_2_ exposure than males [43]. Altered sensitivity to air pollutants may be related to physiological differences because of the hormonal, biological, structural, and morphological differences between men and women, which directly affect the transport of exposed chemicals and the deposition in tissues [44]. In addition, an overall more pronounced immune–inflammatory response in women than in men may also partially explain this result [45]. Our research highlights the need for additional studies to clarify the underlying pathology and mechanism of these discrepancies, and explain the reasons for this phenomenon.

In this study, air pollutants had more short-term effects on IHD hospitalization during cold seasons than warm seasons. A growing number of studies have identified this seasonal difference [25,46]. Our finding echoes a study conducted in Shanghai [7], which found an increased risk associated with air pollution during cold seasons. The reason for this may be due to the seasonal variation in air pollution in Wuhan, in which air pollution has higher concentration in winter, and lower concentration in summer [5]. The higher air pollution levels in the cold season may be the result of a combination of factors, including rising biomass and fossil fuel consumption in China during winter, relatively low temperatures that accelerate the transformation of particulate matter, and low wind speeds that limit the dispersion of air pollutants [47]. Another possible explanation is that the cold temperatures in winter increase individual blood pressure and viscosity, which may contribute to the risk of IHD onset [48]. However, studies showed contrasting results. For example, a multi-city analysis found a stronger association between PM_2.5_ and acute myocardial infarction hospitalizations during warm seasons (April to September) than cold seasons [49]. A study in Beijing also found that the effects of air pollution are more pronounced on warm days than on cool days [38]. These differences could be related to factors such as chemical components, exposure patterns of local populations, population susceptibility, and lifestyle differences [12].

Our subgroup analyses shed some light into modifications in the possible effects of selected comorbidities. We observed that the likelihood of IHD hospitalization among diabetics was higher when exposed to NO_2_ and SO_2_. The same result was observed in patients with hypertension, although the risk estimates were less biased. Consistent with the results of the present study, a study in Brazil found an increase in hospitalizations for cardiovascular disease due to SO_2_ exposure in people with diabetes compared with those without diabetes [50] Similarly, a study conducted in California found that NO_2_ had a greater impact on IHD admissions with a secondary diagnosis of arrhythmia [51]. This result suggests that people with hypertension or diabetes should be alert to low air quality, and take proactive precautions, such as wearing a mask, when going outdoors.

Surprisingly, the possibility of IHD hospitalization was lower when patients with hyperlipidemia were exposed to ambient PM_2.5_, PM_10_, NO_2_, and SO_2_. Similar results were found in patients with hypertension exposed to PM_2.5_ and PM_10_, and in patients with diabetes exposed to PM_10_. Comparable results were found in a study on the relationship between air pollution and stroke hospitalization in northern China, which found that the risk of hospitalization for stroke with hypertension as a comorbidity due to NO_2_ was lower than that without hypertension [52]. Similar results have been found in articles on air pollution-related respiratory disease hazards. For example, a population-based UK Biobank study found that asthma status altered the relationship of PM_2.5_ and NO_2_ with the prevalence of chronic obstructive pulmonary disease, with a remarkably stronger association in non-asthmatic patients [53]. The potential modifications of underlying health conditions, including hypertension, diabetes, and hyperlipidemia, on air pollution-related hazards have been rarely studied. This difference may be related to differences in individual behavioral strategies, as people with these chronic conditions may be more health conscious and less exposed to polluted air [54]. Another possible explanation is that the medications used by people with these complications (against hypertension, diabetes, or hyperlipidemia) provide a protective effect. For example, medications that lower blood pressure have been found to possibly help soften blood vessels and reduce the risk of IHD [55]. More studies are warranted to further investigate this issue to protect vulnerable subpopulations from air pollution. Overall, our study suggests that reducing the air pollution exposure of people without underlying disease is also particularly important for reducing the risk of IHD.

Finding targeted and effective protection measures is essential to reduce the health risks of air pollution. For the individual, we can protect ourselves from air pollution by reducing exposure and decreasing the impact [56]. On the one hand, individuals can reduce unnecessary exposure by wearing masks and using household clean energy [57]. On the other hand, significant lifestyle improvements, including physical activity, reducing smoking and drinking, a nutritious diet, and antioxidant foods supplementation, may contribute to a lower risk of exposure [58,59,60]. For the government, as automobile exhaust is an important source of air pollution in Wuhan, several traffic-related measures, such as car scrappage schemes, and promoting public transport and cycling may be positively desirable [61]. In addition, Wuhan’s current energy structure is unreasonable, with a high proportion of coal-fired power generation. Strengthening total control of coal consumption, retrofitting old boilers, and increasing the efficiency of clean energy may contribute to less air pollution.

Our study has several strengths. First, this study is one of the few studies that reported the hazardous effects of air pollutants on IHD in a metropolitan area in central China. Second, our study included data on all IHD hospitalizations in Wuhan from 2017 to 2018, and, therefore, has a large sample size of 139,616 hospital admissions for IHD. Third, we obtained data on air pollution and the atmospheric environment from reliable sources, and the air quality monitoring stations covered the entire area of Wuhan. Therefore, the air pollution data strongly supported the statistical findings. Finally, we used a GAM and took into account confounding factors, which makes our results more reliable.

Nevertheless, several limitations still exist in the present study. First, we could not measure the air pollution exposure at the individual level because of the lack of available data, such as the distance from the monitoring stations to the individual’s residence or workplace, as well as the unavailability of indoor exposure data. Second, we were unable to investigate the relationship between some potentially sensitive subgroups and air pollution because of the lack of information on important risk factors that may influence an individual’s susceptibility to IHD, such as obesity, smoking, alcohol consumption, diet, and exercise. Further studies are needed to assess individual air exposure in more detail, and individual lifestyle factors should be included to better assess the hazards of air pollution.

## 5. Conclusions

The results show that the increase in the concentrations of air pollutants, including PM_2.5_, PM_10_, NO_2_, and SO_2_, has remarkable associations with the increase in daily IHD hospitalizations, especially during cold seasons and in older people (≥76 years). The associations of IHD hospitalization with PM_2.5_ and PM_10_ appear to be stronger in men than in women, and the opposite was observed between IHD hospitalization and exposure to NO_2_ or SO_2_. People with underlying medical conditions, such as hypertension and diabetes, are more susceptible to the effects of NO_2_ and SO_2_. Our study provides new evidence of the effects of air pollution on the increased incidence of IHD in central China, and acts as an effective reference for local administrative departments to formulate environmental protection policies.

## Figures and Tables

**Table 1:** Population characteristics of study participants (*n* = 139,616).

Population Characteristics	IHD Hospitalizations (*n* = 139,616)
Age, mean ± SD	68.56 ± 12.4
Age group, *n* (%)	
≤65	58,197 (41.7)
66–75	37,351 (26.8)
≥76	44,068 (31.6)
Gender, *n* (%)	
Male	77,724 (55.7)
Female	61,892 (44.3)
Season at admission, *n* (%)	
Cold	71,832 (51.4)
Warm	67,784 (48.6)
With Hypertension, *n* (%)	88,425 (63.3)
With Diabetes, *n* (%)	34,560 (24.8)
With Hyperlipidemia, *n* (%)	24,891 (17.8)

**Table 2:** Summary characteristics of ambient air pollutants and daily IHD hospitalization data in Wuhan, China, 2017–2018.

Variables	Mean ± SD	Min	P25	Median	P75	Max
Air pollutants (μg/m^3^)						
PM_2.5_	48.43 ± 29.47	6.97	26.79	41.51	61.79	214.10
PM_10_	82.07 ± 47.89	9.86	49.15	73.52	10.54	559.35
NO_2_	42.22 ± 16.11	16.67	32.04	41.21	52.16	100.27
SO_2_	9.78 ± 4.80	3.00	6.19	8.57	12.05	30.57
Weather conditions						
Temperature (°C)	17.52 ± 9.32	−3.80	9.50	18.10	25.80	33.90
Humidity (%)	79.39 ± 10.26	47.00	72.00	80.00	87.00	100.00
Daily IHD case	192.60 ± 69.50	47.00	135.00	189.00	238.00	456.00

**Table 3:** Excess risks (%) and 95% CIs of daily IHD hospitalizations for per 10 ug/m^3^ increase in PM_2.5_, PM_10_, NO_2_, and SO_2_ at different lag days.

Air Pollution	Single Lag		Moving-Average Lag	
	Lag Day	ERs (95% CI)	Lag Day	ERs (95% CI)
PM_2.5_	0	0.50 (0.27–0.73)	01	0.31 (0.18–0.44)
	1	0.49 (0.25–0.73)	02	0.20 (0.05–0.34)
	2	0.03 (−0.19–0.26)	03	0.24 (0.09–0.39)
	3	0.03 (−0.19–0.26)		
PM_10_	0	0.50 (0.36–0.64)	01	0.55 (0.41–0.70)
	1	0.39 (0.27–0.52)	02	0.48 (0.32–0.63)
	2	0.09 (−0.02–0.21)	03	0.38 (0.21–0.54)
	3	−0.10 (−0.22–0.02)		
NO_2_	0	2.86 (2.45–3.26)	01	2.76 (2.31–3.20)
	1	1.78 (1.38–2.18)	02	2.43 (1.95–2.91)
	2	0.60 (0.21–0.99)	03	2.06 (1.55–2.56)
	3	−0.2 (−0.59–0.19)		
SO_2_	0	3.82 (2.17–5.49)	01	2.17 (0.45–3.93)
	1	−0.03 (−1.46–1.42)	02	2.20 (0.41–4.02)
	2	1.37 (0.01–2.75)	03	1.49 (−0.36–3.37)
	3	−0.77 (−2.1–0.58)		

## Data Availability

Not applicable.

## Appendix A

**Table A1:** Distribution of daily air pollutant concentrations according to China ambient air quality standards (AQS), 2017–2018.

Air Pollutants	China AQS Grade I Criteria		China AQS Grade II Criteria	
	24-h Concentration (μg/m^3^)	Days Exceeded Criteria (%)	24-h Concentration (μg/m^3^)	Days Exceeded Criteria (%)
PM_2.5_	35	442 (61)	75	115 (16)
PM_10_	50	539 (74)	150	44 (6)
NO_2_	80	31 (4)	80	31 (4)
SO_2_	50	0 (0)	150	0 (0)
